# Laccase Production and Differential Transcription of Laccase Genes in *Cerrena* sp. in Response to Metal Ions, Aromatic Compounds, and Nutrients

**DOI:** 10.3389/fmicb.2015.01558

**Published:** 2016-01-12

**Authors:** Jie Yang, Guozeng Wang, Tzi Bun Ng, Juan Lin, Xiuyun Ye

**Affiliations:** ^1^Fujian Key Laboratory of Marine Enzyme Engineering, Fuzhou UniversityFuzhou, China; ^2^Faculty of Medicine, School of Biomedical Sciences, The Chinese University of Hong KongHong Kong, China

**Keywords:** *Cerrena* sp., qPCR, laccase, differential regulation, promoter

## Abstract

Laccases can oxidize a wide range of aromatic compounds and are industrially valuable. Laccases often exist in gene families and may differ from each other in expression and function. Quantitative real-time polymerase chain reaction (qPCR) was used for transcription profiling of eight laccase genes in *Cerrena* sp. strain HYB07 with validated reference genes. A high laccase activity of 280.0 U/mL was obtained after submerged fermentation for 5 days. Laccase production and laccase gene transcription at different fermentation stages and in response to various environmental cues were revealed. HYB07 laccase activity correlated with transcription levels of its predominantly expressed laccase gene, *Lac7*. Cu^2+^ ions were indispensable for efficient laccase production by HYB07, mainly through *Lac7* transcription induction, and no aromatic compounds were needed. HYB07 laccase synthesis and biomass accumulation were highest with non-limiting carbon and nitrogen. Glycerol and inorganic nitrogen sources adversely impacted *Lac7* transcription, laccase yields, and fungal growth. The present study would further our understanding of transcription regulation of laccase genes, which may in turn facilitate laccase production as well as elucidation of their physiological roles.

## Introduction

White-rot fungi secrete ligninolytic enzymes such as lignin peroxidases, manganese peroxidases, versatile peroxidases, and laccases (Wong, 2009). Laccases comprise a class of multi-copper containing oxidases that are environmentally friendly and industrially important. Laccases have low substrate specificity and can oxidize a wide range of substrates such as phenolic lignin compounds, recalcitrant dyestuffs, and other environment pollutants, accompanied by concomitant reduction of molecular oxygen to water. Laccases have found applications in food, bioremediation, textile, biofuel, and other industries (Giardina et al., 2010).

Unraveling gene expression patterns is important for elucidating gene function as well as metabolic engineering to improve yields of desired products. Northern blotting, semi-quantitative reverse transcription-polymerase chain reaction (RT-PCR), and quantitative real-time PCR (qPCR) have been used to determine mRNA abundance. Laccase production, often at the level of transcription, responds to various environmental signals such as metal ions, aromatic compounds, and carbon and nitrogen sources and concentrations (Piscitelli et al., 2011; Janusz et al., 2013). The changes in laccase gene expression levels are also likely related to their function during the fungal life cycle (Solé et al., 2012). However, only a few laccase expression studies used qPCR, and most of them focused on one or a few laccase genes. Fungal laccases usually exist in gene families, and the family members have different biochemical properties and responses to inducers and nutrients (Giardina et al., 2010).

qPCR is a powerful tool in expression quantification and has gained popularity because it is fast, sensitive, accurate, and cost-effective. Recently, transcription of the laccase gene family in *Pleurotus ostreatus* has been profiled (Castanera et al., 2012, 2013; Pezzella et al., 2012). On the other hand, when it comes to other white-rot fungi, few comprehensive qPCR analyses on the laccase gene families are available, even though the induction response can be gene- and strain-specific (Piscitelli et al., 2011; Janusz et al., 2013). Furthermore, many existing qPCR reports use housekeeping gene(s) for data normalization without prior validation, which can lead to erroneous interpretation of the results (Kozera and Rapacz, 2013).

The genus *Cerrena* has recently attracted research attention with its potentials in laccase production and applications (D'souza et al., 2006; Michniewicz et al., 2006; Elisashvili et al., 2010; Lisova et al., 2010; Kucharzyk et al., 2012; Songulashvili et al., 2012). Despite sequencing of *C. unicolor* genome (http://www.jgi.doe.gov), expression regulation of *Cerrena* laccase genes remains largely unknown. We have isolated a white-rot fungus *Cerrena* sp. strain HYB07 with high laccase yields in a short production cycle. Two HYB07 laccases (i.e., Lac1 and 7) have been characterized; they have different pH and temperature optima, substrate ranges and catalytic activity (Yang et al., 2014, 2015). In the present study, transcription of eight laccase genes in *Cerrena* sp. HYB07 at different phases of submerged fermentation and in response to various inducers (metal ions and aromatic compounds), carbon/nitrogen ratios and carbon and nitrogen sources was investigated. The present work will provide a theoretical foundation for elucidating the complex molecular mechanism underlying laccase expression regulation in *Cerrena* sp. HYB07 and pave the way for its commercialization and utilization.

## Materials and methods

### Strain, culturing conditions, and sample preparation

*Cerrena* sp. strain HYB07 (Yang et al., 2014) was maintained on potato dextrose agar (PDA) (Difco, USA) at 4°C in the culture collection of Fuzhou University, China. For laccase fermentation, 5 mycelial plugs (1 cm diameter) were removed from the peripheral region of 4-days-old PDA plate stored at 30°C and inoculated in 50 mL potato dextrose broth (PDB) seed medium in a 250 mL Erlenmeyer flask. After growing for 2 days at 30°C and 200 rpm, an aliquot was transferred to a second PDB medium at the ratio of 8% (v/v). After another 2 days, the fermentation medium (50 mL in 250 mL Erlenmeyer flasks) was inoculated with the second seed culture at the concentration of 8% (v/v). Unless otherwise stated, the fermentation medium contained (g L^−1^): maltodextrin, 60; peptone, 10; ammonium tartrate, 1.6; KH_2_PO_4_, 6; MgSO_4_·7H_2_O, 4.14; CaCl_2_, 0.3; NaCl, 0.18; CuSO_4_·5H_2_O, 0.0625; ZnSO_4_·7H_2_O, 0.018; and vitamin B1 0.015. This medium was also referred to as the control, regular or HNHC (high nitrogen and high carbon) medium in this manuscript.

To test the effects of inducers on laccase production, Cu^2+^ or Zn^2+^ ions were not added to the fermentation medium; aromatic compounds, including 2,2′-azino-bis(3-ethylbenzothiazoline-6-sulfonic acid) (ABTS), caffeic acid, ferulic acid, guaiacol, p-hydroxybenzoic acid, nicotinic acid, syringic acid, and vanillic acid, were individually added at a final concentration of 0.5 mM. ABTS was purchased from Sigma-Aldrich (USA), and all other aromatic compounds were purchased from Sinopharm Chemical Reagent Co., Ltd (China) or Aladdin (China). For media with different carbon/nitrogen ratios, HNLC (high nitrogen and low carbon) medium contained 6 g dextrin instead of 60 g; LNHC (low nitrogen and high carbon) contained 1 g peptone and 0.16 g ammonium tartrate instead of 10 and 1.6 g, respectively; LNLC (low nitrogen and low carbon) medium contained 6 g dextrin, 1 g peptone, and 0.16 g ammonium tartrate. For media with different carbon sources, dextrin was replaced with glucose (monosaccharide), fructose (monosaccharide), maltose (disaccharide), sucrose (disaccharide), or glycerol at the same concentration of 6%. For media with alternative nitrogen sources, peptone was substituted with 10 g NH_4_NO_3_ or ammonium tartrate, while 1.6 g ammonium tartrate in the original medium recipe remained the same. Since our preliminary experiments showed that addition of aromatic compounds on day 0 (the time of culture inoculation) impaired fungal growth, aromatic compounds were added on day 2; all other treatments started from day 0. Laccase activity was measured on day 6. For each treatment, two independent experiments were carried out with three replicates for each experiment.

Subsequently, selected samples were analyzed with qPCR. For growth phase experiments, mycelia were harvested every 24 h from days 1 to 6. For ABTS and guaiacol treatments, control and treated fungal cultures were taken on the fourth day of fermentation. For all other treatments, the control and treated cultures were collected on the second day, when biomass reached maximum and extracellular laccase activity was on exponential increase. These other treatments included: media with no additional Cu^2+^ or Zn^2+^, various carbon/nitrogen ratios (HNLC, LNHC, and LNLC) and alternative carbon and nitrogen sources (i.e., glucose, sucrose, glycerol, NH_4_NO_3_ and ammonium tartrate).

### Enzyme activity assay and fungal dry mass determination

Extracellular laccase activity was spectrophotometrically assayed at 30°C and pH 3.0 with ABTS at 420 nm. One unit of enzyme activity was defined as the amount of enzyme needed to oxidize 1 μmol ABTS in 1 min. All measurements were carried out in triplicate.

For biomass measurements, triplicate cultures were harvested at the same time as for qPCR analysis. Mycelia were filtered with pre-dried Whatman filter paper, dried in an oven at 70°C for 24 h and weighed.

### RNA extraction and reverse transcription

Mycelia from three biological replicates were pooled, and total RNA was extracted with Trizol (Life Technologies, USA) according to the manufacturer's instructions. RNA quantity was measured with a NanoDrop 2000 UV-Vis spectrophotometer (Thermo Scientific, USA). The ratios of the absorbance at 260 and 280 nm of the samples were between 1.8 and 2.0. RNA integrity was checked by 1% agarose gel electrophoresis before reverse transcription (data not shown).

Contaminating genomic DNA was removed and reverse transcription was carried out by using TransScript One-Step gDNA Removal and cDNA Synthesis SuperMix (Transgen, China). Each reaction contained 2 μg total RNA, 0.5 μg oligo-(dT)_12−18_ primer and 1 μM 18S-rRNA-specific primer (the reverse qPCR primer for *18S rRNA*) (Zhu and Altmann, 2005; Yang et al., in press).

### Primers

qPCR primers for the eight laccase genes (*Lac1*-*8*) and housekeeping genes were designed by PrimerQuest (Integrated DNA Technologies, USA; Table 1). All qPCR primer pairs were specific, as determined by agarose gel electrophoresis (data not shown) and melting curves (Supplementary Figure S1) of qPCR products. Amplification efficiencies of the primer pairs were calculated from the standard curves of serial 1:10 dilutions of DNA templates (cDNA or recombinant plasmids containing the target gene fragments). The amplification efficiencies were 91.2–100.7% (*R*^2^≥0.99), and the amplicon sizes ranged from 91–220 bp.

**Table 1:** **qPCR primers for the eight laccase genes and seven housekeeping genes in ***Cerrena*** sp. HYB07**.

**Gene**	**GenBank #**	**Primer sequence(5′ → 3′)**	**Amplicon length (bp)**	**Amplification efficiency (%)**	***R*^2^**
*Lac*1	KC540913	Forward: CTTGGTTCCTCCACTGTCATATC	116	95.34	0.997
		Reverse: GTTATTCCAGGACTCAGGAACAG			
*Lac*2	KF317944	Forward: GGCCAAACTGGTTACAATTTCA	114	97.51	0.996
		Reverse: GAACCAAGGTCCAGGGTTATC			
*Lac*3	KF317945	Forward: CACATCGACTGGCATTTGGA	93	92.29	0.999
		Reverse: GTCAGCAGGGATGTTAGTGTTAG			
*Lac*4	KF317946	Forward: CGGGCAAACCACATACAACTA	105	98.02	0.999
		Reverse: CCGGGATTATCGGTCACAAATC			
*Lac*5	KF317947	Forward: ACATTGACTGGCACTTGGA	91	91.18	0.998
		Reverse: CAGTCCTTAGGTGTTGGGTTAG			
*Lac*6	KF317943	Forward: CGTTAGGGACGTGGTGAATATC	107	96.32	0.994
		Reverse: CGATATGGCAGTGGAGGAAC			
*Lac*7	KF317949	Forward: CTGGTCAAACTACTCCCAACTAC	95	94.43	0.998
		Reverse: GGTGGTGAAACGGATGGTAA			
*Lac*8	KF317948	Forward: CAGGAGAGACCACCTACAATTATG	101	93.18	0.999
		Reverse: GTTGTCAGTAGTGAAGCGGATAG			
*18S rRNA*	KM233493	Forward: AGACGGAAGTTTGAGGCAATAA	105	95.86	0.997
		Reverse: CTTCCGGCCAAGGTGAATAA			
*ATP*6	KP099099	Forward: CAAGAGCTAATGGAGTACCTGAA	96	93.83	0.994
		Reverse: CACTATATGGACGGCTGTTACT			
*Cyt-c*	KP099098	Forward: CTGATATGGCCTTCCCTAGATTG	110	92.04	0.991
		Reverse: CATCCTGTACCAGCTCCATTT			
β*-tubulin*	KP099103	Forward: TTAGGTCGCCACTATCTTCCG	220	100.67	0.998
		Reverse: AACTGGTCGCTGACACGCT			
*GAPDH*	KP099102	Forward: CCGAGTACTTGGAGTCGTATTG	92	97.84	0.999
		Reverse: TGCCAAGAAGGTCATCATCTC			
*RPB2*	KP099100	Forward: GTATGGTTTGTCCTGCTGAAAC	95	93.84	0.996
		Reverse: GAGAACGAACCGACGGAAATA			
*TEF1*	KP099101	Forward: CTACCAACGTGACCACTGAA	102	94.75	0.993
		Reverse: GACGTTCTTGACGTTGAAACC			

### qPCR

qPCR was performed on an Applied BioSystems 7500 Real-Time PCR system (Life Technologies, USA). Each reaction mixture (20 μL) contained 10 μL 2 × TransStart Top Green qPCR SuperMix (Transgen, China), 0.4 μL 50 × Passive Reference DyeII, forward and reverse primers (each at 200 nM), and 2 μL cDNA (1:50 diluted). Amplification was carried out as follows: an initial denaturation step of 94°C for 30 s, followed by 40 cycles of 94°C for 5 s and 60°C for 34 s. All reactions were performed in triplicate. RT negative controls (containing RNA template without reverse transcription) were run for every sample by using *18S rRNA* as the target to check for DNA presence. No-template negative controls were included every run to detect possible contamination or carryover.

The 2^−▵▵Ct^ relative quantification strategy (Livak and Schmittgen, 2001; Schmittgen and Livak, 2008) was employed to calculate relative quantities of the laccase transcripts under various experimental conditions by using the ExpressionSuite v1.0.3 (Life Technologies, USA). Validated reference genes were used for data normalization.

### Reference gene selection

Expression stability of seven candidate reference genes, including *18S rRNA, ATP6* (ATP synthase 6), β-*tubulin, Cyt-c* (cytochrome c oxidase subunit 1), *GAPDH* (glyceraldehyde 3-phosphate dehydrogenase), *RPB2* (RNA polymerase II second largest subunit), and *TEF1* (translation elongation factor 1-alpha), under various experimental conditions were evaluated with geNorm (Vandesompele et al., 2002). geNorm defines the stability index *M* as the average pairwise variation of a particular gene with all other candidate genes, and *M*-values below 1.5 indicate high expression stability. Stability ranking is achieved through stepwise exclusion of the most variable gene, and the *M*-values for the remaining genes are recalculated. The two most stable genes cannot be ranked in order since gene ratios are used to measure gene stability. geNorm also determines minimum number (*n*) of reference genes based on pairwise variation (*V*_*n*∕*n*__+1_) analysis between the normalization factors NF_*n*_ and NF_*n*__+1_. The recommended cutoff value (0.15) was adopted, so the minimal number of reference genes was decided from the first *V*_*n*∕*n*__+1_ value lower than 0.15.

Samples were divided into five groups for reference gene selection. Group A consisted of six samples collected on days 1 to 6; Group B consisted of six samples under different induction conditions (no Cu^2+^, no Zn^2+^, ABTS, guaiacol, and the respective controls); Group C consisted of four samples cultivated under different carbon/nitrogen ratios (HNHC, HNLC, LNHC, and LNLC); Group D consisted of six samples cultivated under different carbon or nitrogen sources (glucose, sucrose, glycerol, NH_4_NO_3_, ammonium tartrate, and control); Group E contained all the samples. Groups C and D shared the same 2nd-day control sample fermented in the regular medium, which was also a control for Group B in addition to a 4th-day control (for aromatic compound treatment). Samples were grouped for laccase gene transcription analysis based on selected reference genes.

## Results

### Laccase production by *cerrena* sp.

**Figure 1:**
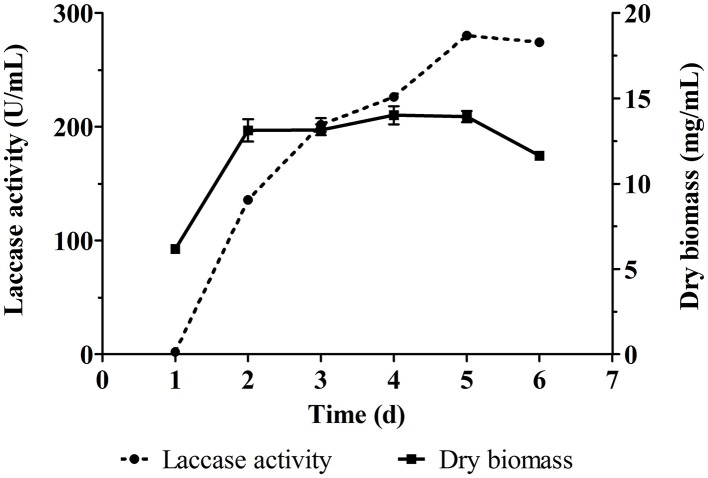
**Laccase production and biomass accumulation of ***Cerrena*** sp. HYB07**. Fermentation was carried out in 250 mL Erlenmeyer flasks at 30°C and 200 rpm. The fermentation medium (50 mL) was inoculated with the second seed culture at the concentration of 8% (v/v). The fermentation medium contained (g L^−1^): maltodextrin, 60; peptone, 10; ammonium tartrate, 1.6; KH_2_PO_4_, 6; MgSO_4_·7H_2_O, 4.14; CaCl_2_, 0.3; NaCl, 0.18; CuSO_4_·5H_2_O, 0.0625; ZnSO_4_·7H_2_O, 0.018; and vitamin B1 0.015.

*Cerrena* sp. HYB07 is a white-rot fungal strain with high laccase yields and a short production cycle (Yang et al., 2014). The fermentation medium used in this study resulted in higher laccase yields (280.0 U/mL) than previously used PDY medium (210.8 U/mL). The strain showed a linear increase in biomass during the first 2 days and then entered stationary phase of growth (Figure 1). Laccase production of *Cerrena* sp. HYB07 in response to various metal ions, aromatic compounds, carbon/nitrogen ratios and nutrients was investigated.

### Effects of metal ions on laccase production

**Figure 2:**
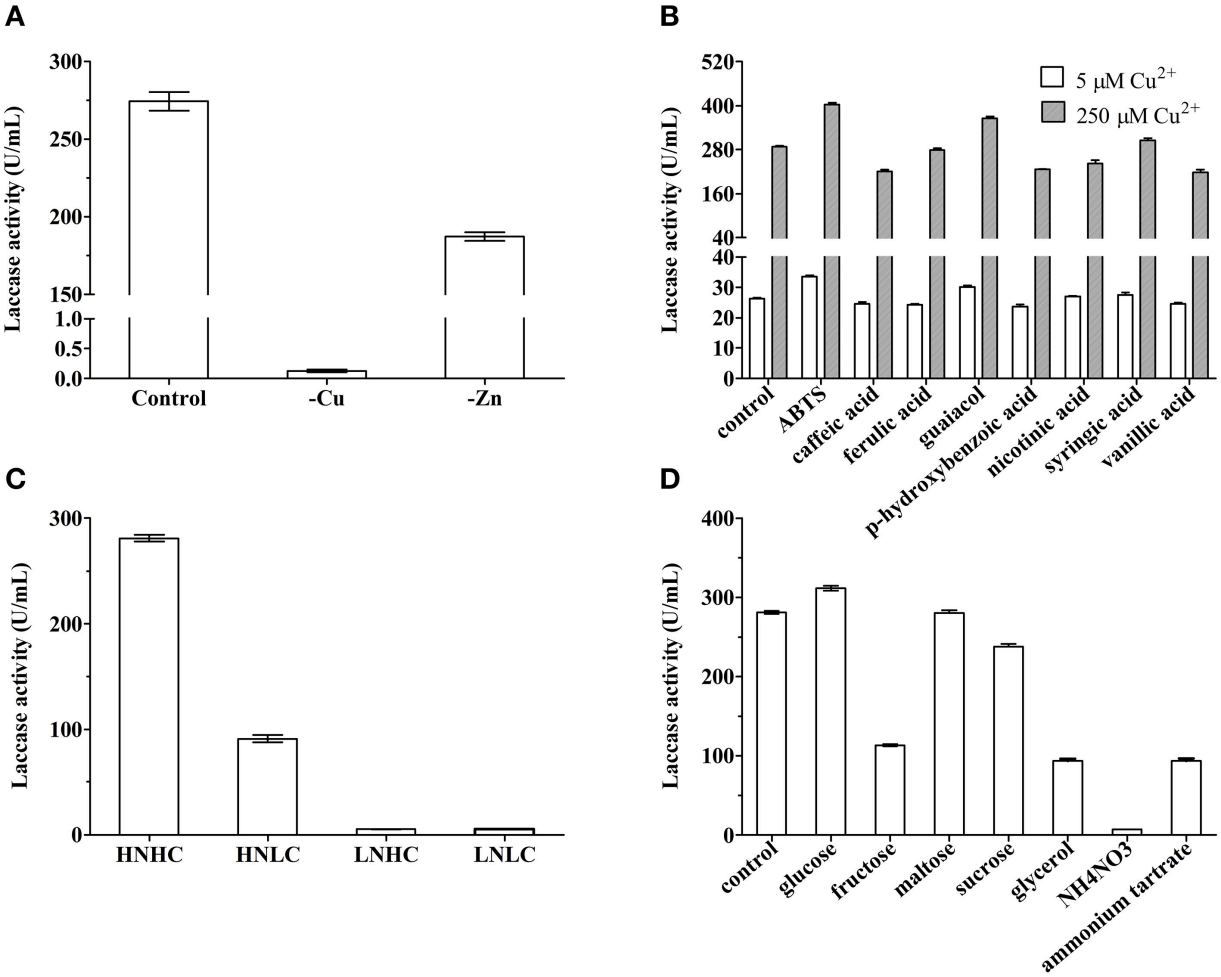
**Laccase production of ***Cerrena*** sp. HYB07 in response to metal ions (A), aromatic compounds (B), carbon/nitrogen ratios (C), and nutrient types (D)**. To test the effects of inducers on laccase production, Cu^2+^ or Zn^2+^ ions were not added to the fermentation medium; aromatic compound ABTS, caffeic acid, ferulic acid, guaiacol, p-hydroxybenzoic acid, nicotinic acid, syringic acid, or vanillic acid, was added at a final concentration of 0.5 mM. For media with different carbon/nitrogen ratios, quantities of carbon and nitrogen sources were alternatively or simultaneously reduced to 10% of the levels in the control medium. For media with different nutrient sources, dextrin was replaced with glucose, fructose, maltose, sucrose or glycerol, and peptone was substituted with 10 g NH_4_NO_3_ or ammonium tartrate. Aromatic compounds were added on day 2 of fermentation, whereas all other treatments started from day 0. Extracellular laccase activity was measured on day 6.

Our preliminary work showed that MnSO_4_, CoCl_2_, FeSO_4_, AlK(SO_4_)_2_, NaMoO_4_, and H_3_BO_3_ did not influence or negatively influenced laccase production, whereas Cu^2+^ and Zn^2+^ stimulated laccase production. Indeed, when Cu^2+^ or Zn^2+^ ions were not added to the fermentation medium, extracellular laccase activity was lowered by 99.95 and 31.78%, respectively (Figure 2A).

### Effects of aromatic compounds on laccase production

Previous work showed that 0.5 mM veratryl alcohol, tannic acid, xylene, phenol, or vanillin failed to improve HYB07 laccase production with 250 μM Cu^2+^. Effects of eight aromatic compounds were tested at two copper concentrations (250 μM as used in the medium and a low copper concentration of 5 μM) (Figure 2B). The highest induction responses were observed with ABTS and guaiacol, which increased laccase production by 40.1 and 26.7% at 250 μM Cu^2+^ and by 28.0 and 15.1% at 5 μM Cu^2+^, respectively. The other aromatic compounds either did not affect or negatively affected laccase production. Overall, laccase yields in the presence of various aromatic compounds were similar regardless of the Cu^2+^ concentration, although the response at the low Cu^2+^ concentration of 5 μM was lower than the response at 250 μM Cu^2+^ concentration.

### Effects of carbon/nitrogen ratios on laccase production

Concentrations and types of carbon and nitrogen nutrients also affected laccase production by *Cerrena* sp. HYB07 (Figures 2C,D). High carbon and nitrogen concentrations in the fermentation medium were beneficial for laccase production (280.0 U/mL), whereas low nitrogen concentrations, regardless of the carbon concentration, dramatically reduced laccase yields to approximately 5.3 U/mL. A laccase activity of 91.0 U/mL, approximately one-third of that of the control medium, was obtained with the HNLC medium (Figure 2C).

### Effects of carbon and nitrogen sources on laccase production

Glucose slightly increased laccase yield to 311.4 U/mL, whereas fructose decreased laccase production by 60% to 113.6 U/mL. Disaccharide maltose did not affect laccase yields, and sucrose lowered laccase yields by 15.5%. Among all carbon sources tested, the lowest laccase activity (93.7 U/mL) was observed with glycerol (Figure 2D). Ammonium nitrate and ammonium tartrate instead of peptone led to laccase production of only 7.1 and 94.8 U/mL, respectively (Figure 2D).

To better understand the mechanism underlying laccase synthesis, qPCR was used to explore the effects of growth phases, inducers, carbon/nitrogen ratios, as well as carbon and nitrogen sources on laccase gene transcription. Based on laccase production studies, the following parameters were selected: (1) days 1–6 of fermentation; (2) no supplementary Cu^2+^ or Zn^2+^; (3) presence of ABTS or guaiacol; (4) alternative carbon source glucose, sucrose or glycerol; (5) alternative nitrogen source NH_4_NO_3_ or ammonium tartrate.

### Reference gene selection

For accurate interpretation of qPCR results, expression stability of seven housekeeping genes, namely *18S rRNA, ATP6, Cyt-c, GAPDH, RPB2, TEF1*, and β-*tubulin* was analyzed with geNorm (Vandesompele et al., 2002). No versatile reference genes were identified for all samples since all *V*_*n*∕*n*__+1_ values of Group E were higher than 0.15 (Supplementary Figure S2). Therefore, the reference genes were selected based on experimental variables: a combination of four references genes (i.e., *Cyt-c, ATP6, TEF1*, and β-*tubulin*) for different growth phases (Group A); *GAPDH* and *Cyt-c* for different induction conditions (Group B); *ATP6* and *TEF1* for various carbon/nitrogen ratios (Group C); and *GAPDH* and *Cyt-c* for various carbon or nitrogen sources (Group D) (Supplementary Figure S2). Next, laccase transcription was analyzed with the respective reference genes.

### Transcription profiling of *cerrena* sp. laccase genes

**Figure 3:**
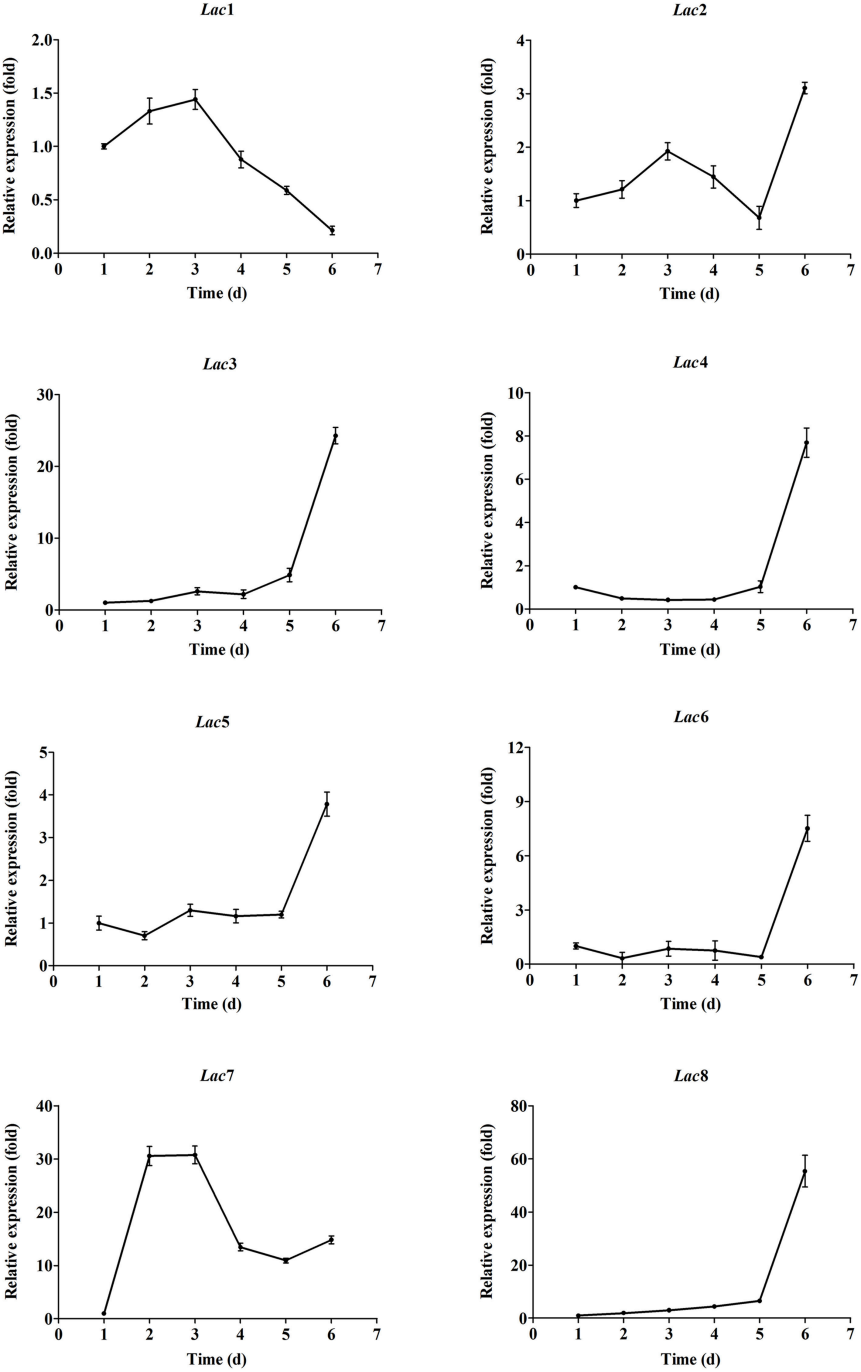
**Expression of the eight laccase genes in ***Cerrena*** sp. HYB07 during submerged fermentation in a 250-mL Erlenmeyer flask**. Transcript levels of each gene on different days are expressed as fold changes compared to the transcript level on day 1. The reference genes used for normalization were *Cyt-c, ATP6, TEF1*, and β*-tubulin*.

**Figure 4:**
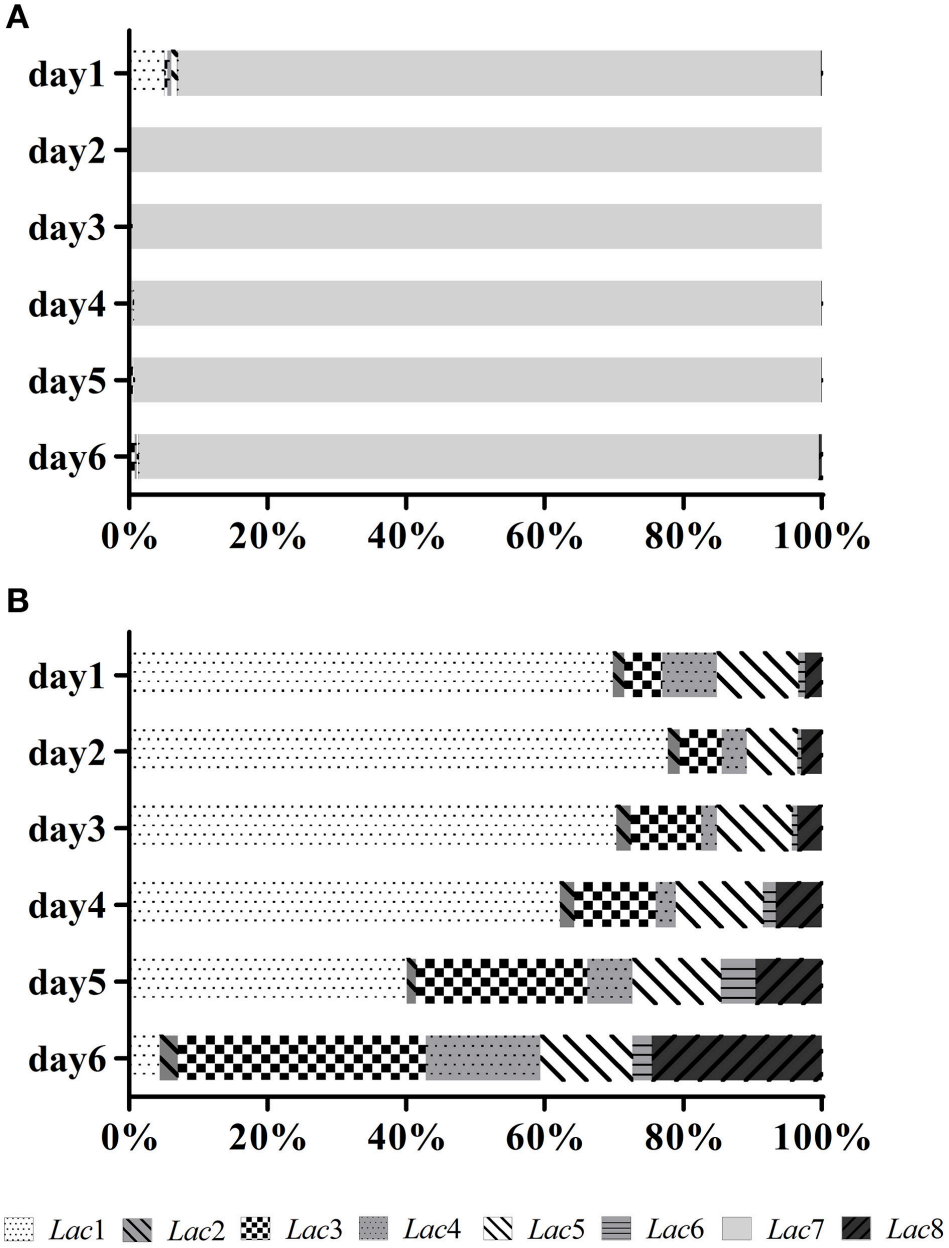
**Relative transcript abundance (expressed as a percentage) of the laccase genes during fermentation**. **(A)** Relative transcript abundance of the eight laccase genes. The total transcript level of all laccase genes was taken as 100%. **(B)** Relative transcript abundance of the seven laccase genes after excluding the predominantly-expressed *Lac7*. The total transcript level of the seven laccase genes was taken as 100%.

Transcription profiles of the eight laccase genes during submerged fermentation are shown in Figure 3. Most significant changes (>10-fold) in transcription levels were observed with *Lac3, 7*, and *8*. Transcription of *Lac3, 4, 5, 6*, and *8* was up-regulated as cells aged; the expression levels on day 6 was 24.3-, 7.7-, 3.8-, 7.5-, and 55.4-fold of the corresponding expression levels on day 1. In contrast, *Lac1* and *7* transcription peaked on days 2-3 and decreased to 0.2- and 14.8-fold on day 6 compared to that on day 1. *Lac2* transcript levels fluctuated throughout the fermentation period. Among the eight laccase genes, *Lac7* was predominantly expressed, accounting for 93–99% of all transcripts of the eight laccase genes (Figure 4A). *Lac1* was the second most abundantly expressed except for day 6, when *Lac3* was the second most abundantly expressed (Figure 4B), but the expression levels of *Lac1* or *3* were negligible relative to those of *Lac7*.

**Figure 5:**
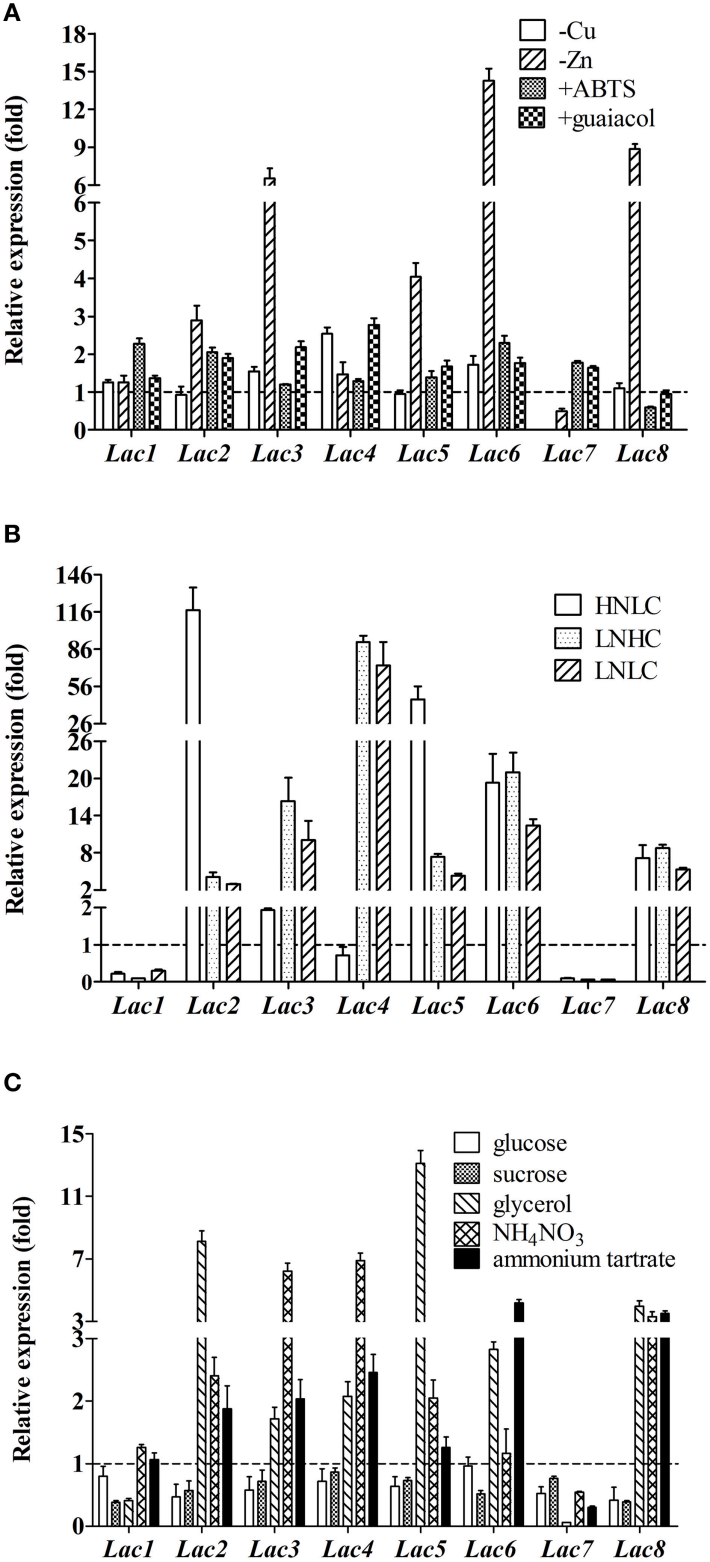
**Relative expression of the eight laccase genes in ***Cerrena*** sp. HYB07 in response to metal ions (A), aromatic compounds (A), carbon/nitrogen ratios (B), and nutrient types (C)**. Transcript levels of each gene in treated samples are expressed as fold changes compared to the transcript level in the control sample (fermented in the control medium) harvested and analyzed at the same time. Treated samples were collected on the second day of fermentation except for aromatic compound-treated samples, which were collected on day 4 (aromatic compounds were added to the fermentation media on day 2). The reference genes used for normalization were as follows: **(A)** *GAPDH* and *Cyt-c* for different induction conditions; **(B)** *ATP6* and *TEF1* for various carbon/nitrogen ratios; **(C)** *GAPDH* and *Cyt-c* for various carbon or nitrogen sources.

When copper ions were not supplemented in the fermentation medium, *Lac7* transcript level was diminished by 1000-fold. Opposite to *Lac7, Lac4* transcription level was higher (2.5-fold) in the absence of Cu^2+^ ions (Figure 5A). *Lac7* was also induced by Zn^2+^ ions, although to a less extent compared to induction by Cu^2+^ ions; its transcript level was reduced by approximately 50% without Zn^2+^ ions. Expression of *Lac2, 3, 5, 6*, and *8* was suppressed by Zn^2+^ ions, with *Lac6* being the most remarkably suppressed (Figure 5A).

ABTS and guaiacol also affected laccase gene transcription (Figure 5A). Approximately two-fold relative expression levels were observed with *Lac1, 2*, and *6* upon ABTS addition. In the presence of guaiacol, *Lac3* and *4* transcription was up-regulated by 2.2- and 2.8-fold. Changes in the expression levels of other laccase genes were less than two-fold.

More dramatic differences were seen with various carbon/nitrogen ratios (Figure 5B). *Lac2* was significantly up-regulated in the HNLC medium by 117.5-fold, followed by *Lac5* (45.6-fold), *Lac6* (19.3-fold), and *Lac8* (7.1-fold). In the two low nitrogen media, similar responses were observed, regardless of the carbon concentration. *Lac4* was most remarkably up-regulated by limiting nitrogen, followed by *Lac6, 3, 8, 5*, and *2*. Low carbon or nitrogen concentrations inhibited expression of *Lac7* (by 11.0- to 16.7-fold) and *Lac1* (by 10.1- to 3.2-fold).

Glucose and sucrose slightly down-regulated transcription of most laccase genes (Figure 5C). Glycerol induced expression of *Lac2, 4, 5, 6*, and *8* by 13.1- to 2.1-fold. On the other hand, glycerol suppressed *Lac7* expression by 17.9-fold; *Lac1* expression was also reduced, albeit to a lesser degree (2.4-fold).

Five laccase genes, namely *Lac2, 3, 4, 5*, and *8*, responded to the inorganic nitrogen source NH_4_NO_3_ with enhanced expression (with *Lac3* and *4* exhibiting highest fold changes of >6).Ammonium tartrate up-regulated transcription of *Lac3, 4, 6*, and *8*. *Lac7* mRNA levels were low with both inorganic nitrogen sources (Figure 5C).

### Putative *cis*-acting responsive elements in laccase gene promoters

Genomatix (http://www.genomatix.de/cgi-bin/eldorado/main.pl) identified multiple putative *cis*-acting transcription regulation sites within the promoter of the eight laccase genes in both orientations, in (Table 2). Promoters of *Lac1, 4, 7*, and *8* had predicted metal response elements (MREs). *Lac6* promoter contained the highest number (4) of ACE1 copper-responsive transcription factor binding sites, followed by 2 in *Lac4* promoter and 1 in *Lac2* and *8* promoters. Promoters of *Lac3* and *5* did not contain any MRE or ACE1 sites. Xenobiotic response elements (XREs) were found in five promoters except for *Lac1, 2*, and *6* promoters.

**Table 2:** **Putative regulatory elements in the promoter regions of ***Cerrena*** sp. HYB07 laccase genes**.

**Gene**	**Length/bp**	**MRE**	**ACE1**	**XRE**	**ARE**	**CSRE**	**MIG**	**RGT**	**CRE**	**NIT2**	**GLN**	**UME6**	**STRE**
*Lac*1	1330	3	0	0	0	4	2	2	9	0	0	1	0
*Lac*2	1397	0	1	0	1	8	0	1	17	0	2	2	2
*Lac*3	825	0	0	1	0	2	0	1	2	0	0	1	1
*Lac*4	2415	1	2	1	0	5	0	0	15	3	0	1	5
*Lac*5	1528	0	0	1	1	4	0	2	13	2	2	0	1
*Lac*6	930	0	4	0	0	3	0	1	2	1	0	1	3
*Lac*7	1544	1	0	1	0	3	0	3	9	1	0	0	0
*Lac*8	3120	2	1	2	2	13	3	2	15	1	3	1	7

*MRE, metal response element; ACE1, ACE1 binding site; XRE, xenobiotic response elements; ARE, antioxidant response element; CSRE, carbon source-responsive element; MIG, MIG binding sites; RGT, Rgt1 binding site; CRE, cAMP responsive element; NIT2, NIT2 binding sites; GLN, Gln3p binding site; UME6, Ume6 binding site; STRE, stress responsive element*.

Carbon- and nitrogen-responsive elements were also identified. All promoters contained various numbers (2–13) of potential carbon source-responsive elements (CSREs; Roth and Schüller, 2001), whereas putative MIG-binding sites were only found in *Lac1* and *8* promoters; zinc finger transcription factor MIG is involved in glucose repression (Lundin et al., 1994). In addition, repressor Rgt1-binding sites were present in the promoters of all laccase except for *Lac4*; Rgt1 binding to DNA is inhibited by glucose (Kim et al., 2003). There were also numerous cAMP-responsive elements in the laccase promoters with possible roles in catabolite-related signaling. Ume6 regulates gene transcription responding to metabolites such as glucose and nitrogen (Williams et al., 2002), and putative Ume6 recognition sites were located in all promoters except for those of *Lac5* and *7*. NIT2 binding sites were identified in five laccase promoters but not those of *Lac1*-*3*, and NIT2 activates gene expression during nitrogen limitation (Fu and Marzluf, 1990). Furthermore, GATA family transcription factor Gln3p binding sites were predicted in *Lac2, 5*, and *8* promoters and might regulate responses of these genes to nitrogen (Coffman et al., 1997). Different types and numbers of putative regulatory elements in the promoter regions suggested differential transcription regulation of these laccase genes.

## Discussion

*Cerrena* sp. HYB07 has been recently reported as a laccase-producing strain with commercial values due to its high laccase yields and short production cycle. Its main laccase, Lac7 (originally named as LacA), has high specific activity, wide substrate specificity and strong decolorization ability (Yang et al., 2014). Laccase production of *Cerrena* sp. HYB07 was regulated by fermentation stages, metal ions, aromatic compounds, carbon/nitrogen ratios, and nutrient types. In order to account for the influence of biomass accumulation on total laccase production, dry biomass-based laccase activity was calculated (Supplementary Figure S3). Although mRNA abundance does not directly translate to protein abundance, dry mass-based laccase activity largely agreed with *Lac7* transcript levels (Supplementary Figures S4, S5), and fluctuations in *Lac7* transcription levels constituted a major cause of fluctuations in laccase yields.

Cu^2+^ ion is the most widely used inducer in laccase production, and it was crucial for high laccase yields of *Cerrena* sp. HYB07. The steep increase in laccase activity upon Cu^2+^ addition was supported by a 1000-fold induction of *Lac7* transcription by Cu^2+^ ions. Other factors may also contribute to high laccase activity in the presence of Cu^2+^ ions. For example, Cu^2+^ ions are needed at the laccase active site (Solé et al., 2012) and may decrease extracellular proteolytic activity (Piscitelli et al., 2011). Of the HYB07 laccase family, *Lac7* was the only gene inducible by Cu^2+^ ions. Zinc ions can substitute copper in transcription activation of certain genes (Collins and Dobson, 1997); *Lac7* mRNA abundance and HYB07 laccase production were increased by Zn^2+^ ions by approximately two-fold, less dramatic than the up-regulation by Cu^2+^ ions.

Aromatic compounds are routinely added to fungal cultures to induce laccase production (Giardina et al., 2010; Piscitelli et al., 2011). For example, syringic acid, among others, enhanced laccase levels of *Trametes velutina* 5930, and the stimulatory effect was synergic with Cu^2+^ ions. In fact, little enzyme activity was detected in cultures not supplemented with any aromatic compound (Yang et al., 2013). Surprisingly, most aromatic compounds tested did not increase HYB07 laccase production regardless of the copper concentration; ABTS and guaiacol only mildly stimulated laccase synthesis. Lack of yield improvements by aromatic compounds was also observed with HYB07 in PDY medium with or without Cu^2+^ supplementation (Supplementary Figure S6). Therefore, laccase synthesis by HYB07 was efficient in the absence of aromatic compounds, which would be beneficial for its commercial production. Similarly, *C. unicolor* VKMF-3196 laccase production needed Cu^2+^ ions, but not aromatic compounds, and an activity of 15 U/mL was achieved after 8-days fermentation (Lisova et al., 2010).

The HNHC medium was most suitable for HYB07 growth and laccase production. Laccase production and biomass accumulation were compromised more severely in the two LN media than in HNLC, implying the importance of sufficient nitrogen in fungal growth and production. Limiting carbon and/or nitrogen reduced *Lac7* transcription level by over 90%, leading to dramatic decreases in laccase activity. Note that up-regulation of other laccase genes might partially compensate for the decreases in *Lac7* transcript levels. Furthermore, different laccase compositions were expected at various carbon/nitrogen ratios except for the two LN conditions with similar laccase transcription patterns (Supplementary Figure S4C). Variations in laccase composition have also been demonstrated in *Coprinus comatus*, which produced six laccase isozymes in the HNLC culture and only two in HNHC, LNHC, and LNLC cultures. In *C. comatus*, however, laccase activity was highest in LNHC medium (Jiang et al., 2013).

Nitrogen source plays a key role in fungal growth and laccase production. It is commonly believed that inorganic nitrogen sources lead to low laccase yields despite sufficient biomass, whereas organic nitrogen sources render high laccase yields (Piscitelli et al., 2011). Indeed, organic nitrogen source peptone favored laccase production and biomass accumulation of HYB07. In contrast, HYB07 fermentation in NH_4_NO_3_ resulted in little biomass and laccase production, as in LN media. Expression profiles of the eight laccase genes with NH_4_NO_3_ also resembled those in LNHC and LNLC samples. We speculated that NH_4_NO_3_ was an inefficient nitrogen source for HYB07, creating a low-nitrogen-like environment. On the other hand, ammonium tartrate allowed for decent fungal growth in spite of low laccase production. It also induced expression of laccase genes responsive to limiting nitrogen, such as *Lac3, 4, 6*, and *8*, and suppressed *Lac7* transcription.

Carbon sources and concentrations have divergent effects on laccase production, depending on the fungal strain (Piscitelli et al., 2011). Glucose repression of laccase expression has been reported for basidiomycete I-62 (Mansur et al., 1998) and *Trametes* sp. AH28-2 (Xiao et al., 2006). In this study, glucose and sucrose (60 g/L) resulted in lower laccase gene transcription levels on day 2, although laccase activity on day 6 was higher in glucose medium. Glycerol was probably not a preferred carbon source for HYB07 and created a low-carbon-like environment, reducing fungal growth and laccase secretion. Laccase genes also behaved similarly with glycerol as with HNLC: *Lac7* and *1* transcription levels were down-regulated, accompanied by significant promotion of *Lac2* and *5* expression. In both cases, *Lac5* transcripts became the second most abundant (Supplementary Figures S4D, 5D).

Putative transcription regulatory elements in the promoter regions of the laccase genes suggested that their expression was regulated. For instance, global transcription regulation of the laccase gene family by carbon and nitrogen was consistent with the presence of various responsive elements implicated in carbon and nitrogen response/repression. *Lac4* expression was responsive to limiting nitrogen, which could be accounted for by three putative NIT2 sites in its promoter (the highest number of the eight laccase promoters). *Lac2* transcription was greatly induced under low carbon conditions; of its eight predicted CSREs, three were adjacent (within the region of -456 to -417 upstream of ATG); this was reminiscent of the distribution pattern of three CSREs in the promoter of *MDH2* (malate dehydrogenase gene 2) required for gluconeogenic growth of *Saccharomyces cerevisiae* (Roth and Schüller, 2001). However, there was not a definite causal relationship between the prediction of an element and the presumed response, or between the number of an element and the intensity of the expected response. For example, *Lac7* was strongly induced by Cu^2+^ ions, but no putative ACE1 copper-responsive transcription factor binding site was identified in its promoter despite the presence of one MRE. It was likely that *Lac7* contained a non-conventional copper-responsive element or that the induction was through a mechanism not involving ACE1 or MREs, as previously proposed (Collins and Dobson, 1997). *Lac4* and *6* contained 2 and 4 potential ACE1 binding sites, respectively, and their expression was either suppressed by Cu^2+^ ions or changed less than two-fold in response to Cu^2+^ ions. Prediction of the responsive elements based on consensus sequences had its limitations and was no doubt an oversimplification of the complex network controlling transcription of laccase genes. Moreover, complex and differential laccase gene transcription patterns were probably the results of a combination of parameters. For example, as the cells age, oxidative stress is accumulated (Si and Cui, 2013), accompanied by depletion of carbon and nitrogen nutrients. Laccase expression could also be regulated by factors including cAMP (Crowe and Olsson, 2001), heat shock (Wang et al., 2012), and calmodulin (Suetomi et al., 2015).

We selected the appropriate reference genes for laccase gene transcription profiling. Housekeeping genes are commonly used as normalization references in relative quantification qPCR experiments, although they may show expression variability with different experimental factors (Kozera and Rapacz, 2013; Castanera et al., 2015). For example, *GAPDH* is one of the most widely used reference genes, but it was unstable with various carbon/nitrogen sources in this study. Therefore, it was necessary to select stable reference genes for accurate data interpretation. Although, it would be ideal to use the same reference genes for all experimental conditions, we could not find such versatile reference genes and therefore divided samples based on experimental variables for reference gene selection and data analysis. This strategy of evaluating candidate reference genes for grouped as well as combined samples is commonly used. Whether versatile reference genes could be identified depended on the organism, candidate genes, and experimental conditions (Zhao et al., 2011; Kianianmomeni and Hallmann, 2013; Kozera and Rapacz, 2013; van Rijn et al., 2014). In order to further illustrate the importance of choosing stable reference genes, we compared laccase gene expression data under HNLC conditions normalized with two choices of reference genes: (1) *ATP6* and *TEF1* (as selected by geNorm), and (2) *GAPDH*. Contradicting or overestimation of the expression values were obtained by the two normalization strategies (Supplementary Figure S7), reinforcing the need to validate reference genes before each gene expression analysis.

## Conclusions

The present study provided insights into laccase production by *Cerrena* sp. HYB07. Cu^2+^ ions, carbon/nitrogen ratios and certain nutrient types exerted remarkable influence on its laccase yields. *Lac7* was the main laccase gene under expression throughout the fermentation cycle, and its transcript abundance was correlated with laccase yields under various experimental conditions. *Lac7* transcription was up-regulated by copper and zinc ions and down-regulated by scarcity of nitrogen and carbon. The advantages of *Cerrena* sp. HYB07 for industrial production were manifested. Efficient laccase synthesis by HYB07 did not require toxic xenobiotic compounds. Furthermore, high expression levels of *Lac7* under high carbon and high nitrogen conditions allow for accumulation of fungal biomass for laccase production. More research is needed to experimentally validate functionality of the putative *cis*-acting regulatory elements in the laccase promoters and decipher the complex regulatory network underlying laccase expression. Future work can also be directed to engineer the strong *Lac7* promoter to drive expression of other, including laccase, genes.

## Author contributions

JY, JL, and XY designed the research, JY and GW performed the experiments, JY and TN analyzed the data and wrote the manuscript, JL and XY contributed reagents.

### Conflict of interest statement

The authors declare that the research was conducted in the absence of any commercial or financial relationships that could be construed as a potential conflict of interest.
