# Supplementary material for: Laccase Production and Differential Transcription of Laccase Genes in Cerrena sp. in Response to Metal Ions, Aromatic Compounds, and Nutrients
Source: Front Microbiol. 2016 Jan 12;6:1558. doi: 10.3389/fmicb.2015.01558 (PMC4710055; doi:10.3389/fmicb.2015.01558)

Supplementary data

for

Laccase production and differential transcription of laccase genes in *Cerrena* sp. in response to metal ions, aromatic compounds and nutrients

Jie Yang[1], Guozeng Wang[1], Tzi Bun Ng[2], Juan Lin[1*], Xiuyun Ye[1*]

[1]Fujian Key Laboratory of Marine Enzyme Engineering, Fuzhou University, Fuzhou, Fujian, China

[2]School of Biomedical Sciences, Faculty of Medicine, The Chinese University of Hong Kong, Shatin, New Territories, Hong Kong, China

Correspondence:

Dr. Juan Lin
Email: ljuan@fzu.edu.cn

Dr. Xiuyun Ye
Email: xiuyunye@fzu.edu.cn

Fig. S1 Melting curves of the laccase gene and housekeeping gene amplicons.

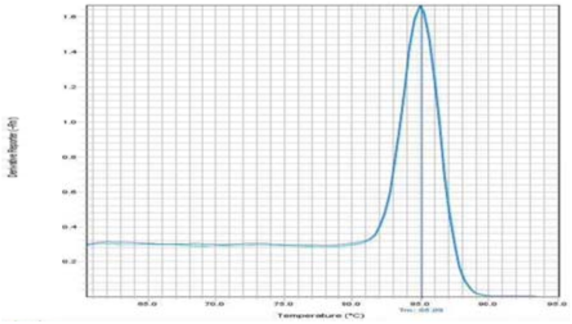

*Lac*1 (Tm = 85.09 ºC)

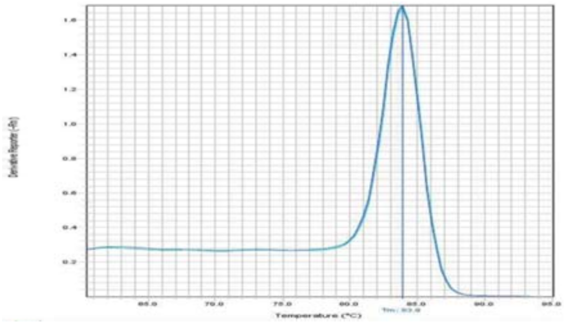

*Lac*2 (Tm = 83.90 ºC）

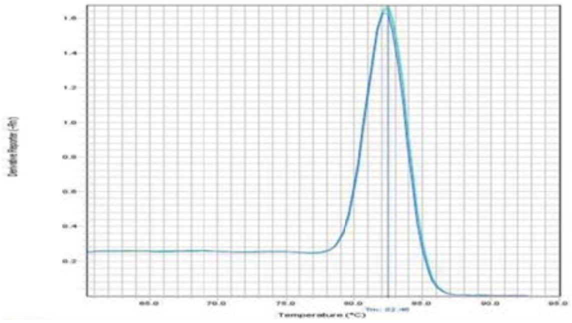

*Lac*3 (Tm = 82.46 ºC）

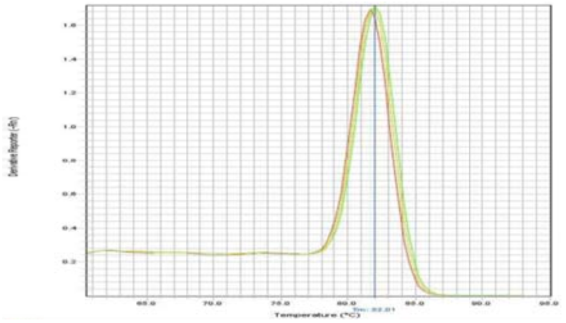

*Lac*4 (Tm = 82.01 ºC）

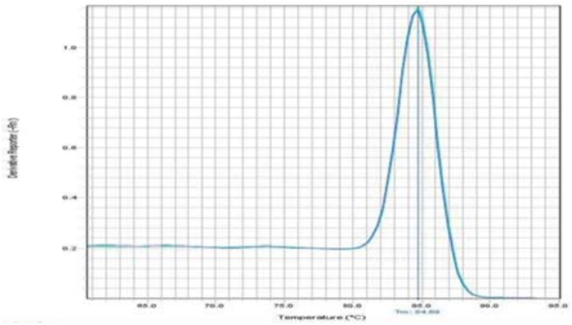

*Lac*5 (Tm = 84.69 ºC）

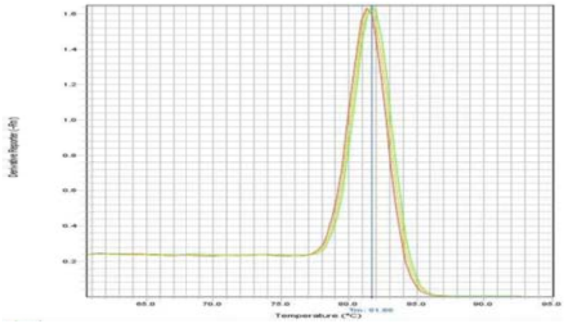

*Lac*6 (Tm = 81.68 ºC）

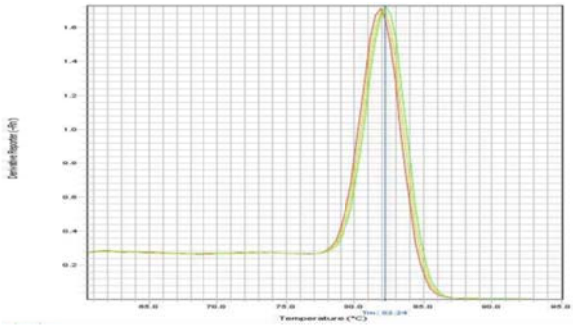

*Lac*7 (Tm = 82.24 ºC)

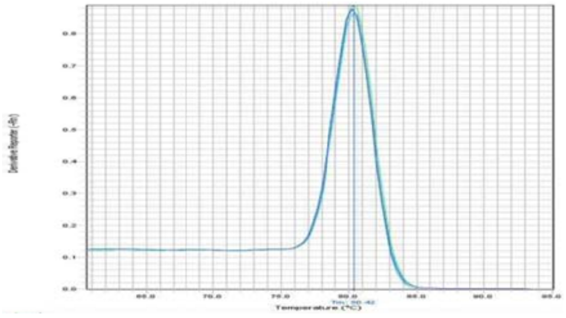

*Lac*8 (Tm = 80.42 ºC)

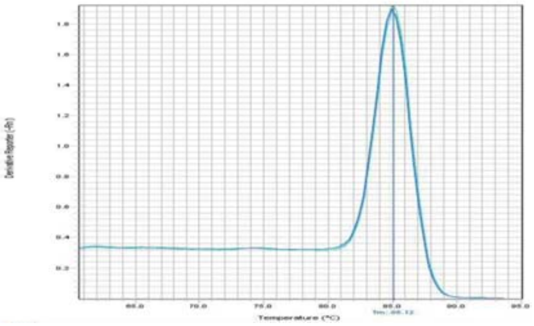

18S rRNA (Tm = 85.12 ºC)

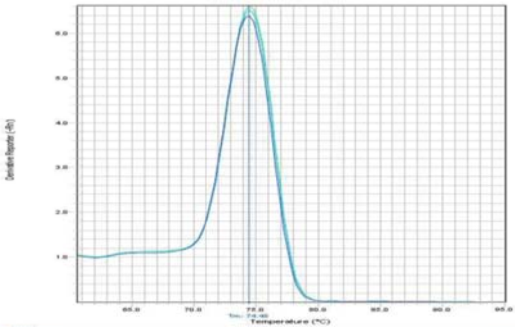

ATP6 (Tm = 74.48 ºC)

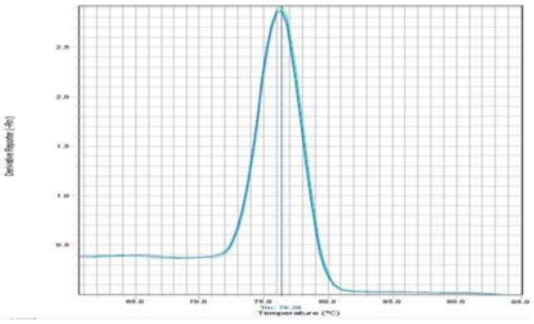

Cyt-c (Tm = 76.36 ºC)

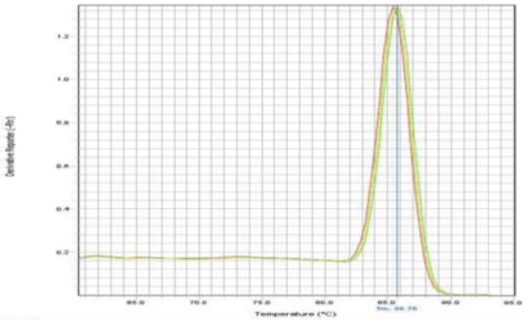

β-tubulin (Tm = 85.78 ºC)

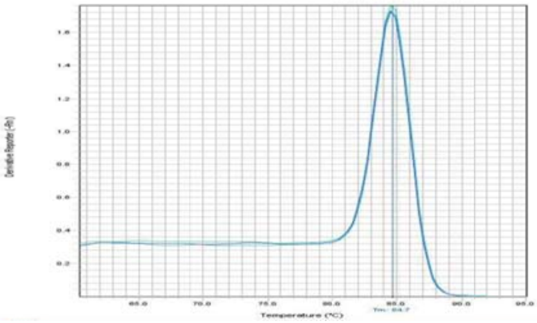

GAPDH (Tm = 84.70 ºC)

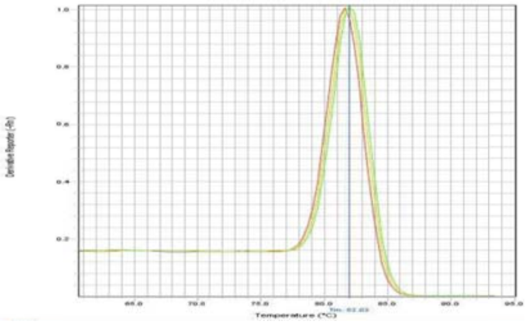

RPB2 (Tm = 82.02 ºC)

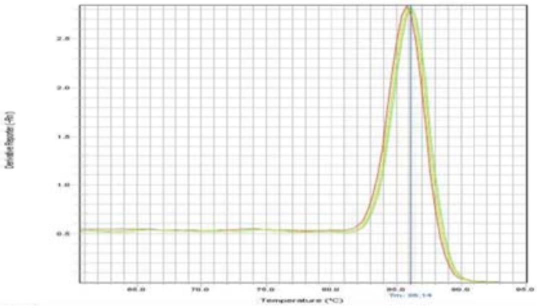

TEF1 (Tm = 86.14 ºC)

Fig. S2 geNorm stability analysis of the seven candidate reference genes for qPCR data normalization. (A)-(E) Average *M* values of the remaining candidate reference genes during stepwise exclusion of the least stable gene in Groups A, B, C, D and E. Group A: samples at different growth phases; Group B: samples under different induction conditions; Group C: samples under different carbon/nitrogen ratios; Group D: samples under different carbon or nitrogen sources; Group E: all samples of Groups A-D. The *x* axis indicates the gene with the highest *M* value in the remaining genes, namely the gene to be excluded for the next round of exclusion (except for the rightmost point). (F) Pairwise variation ($V_{n/n+1}$) analysis to determine the minimal number of reference genes for different sample groups.

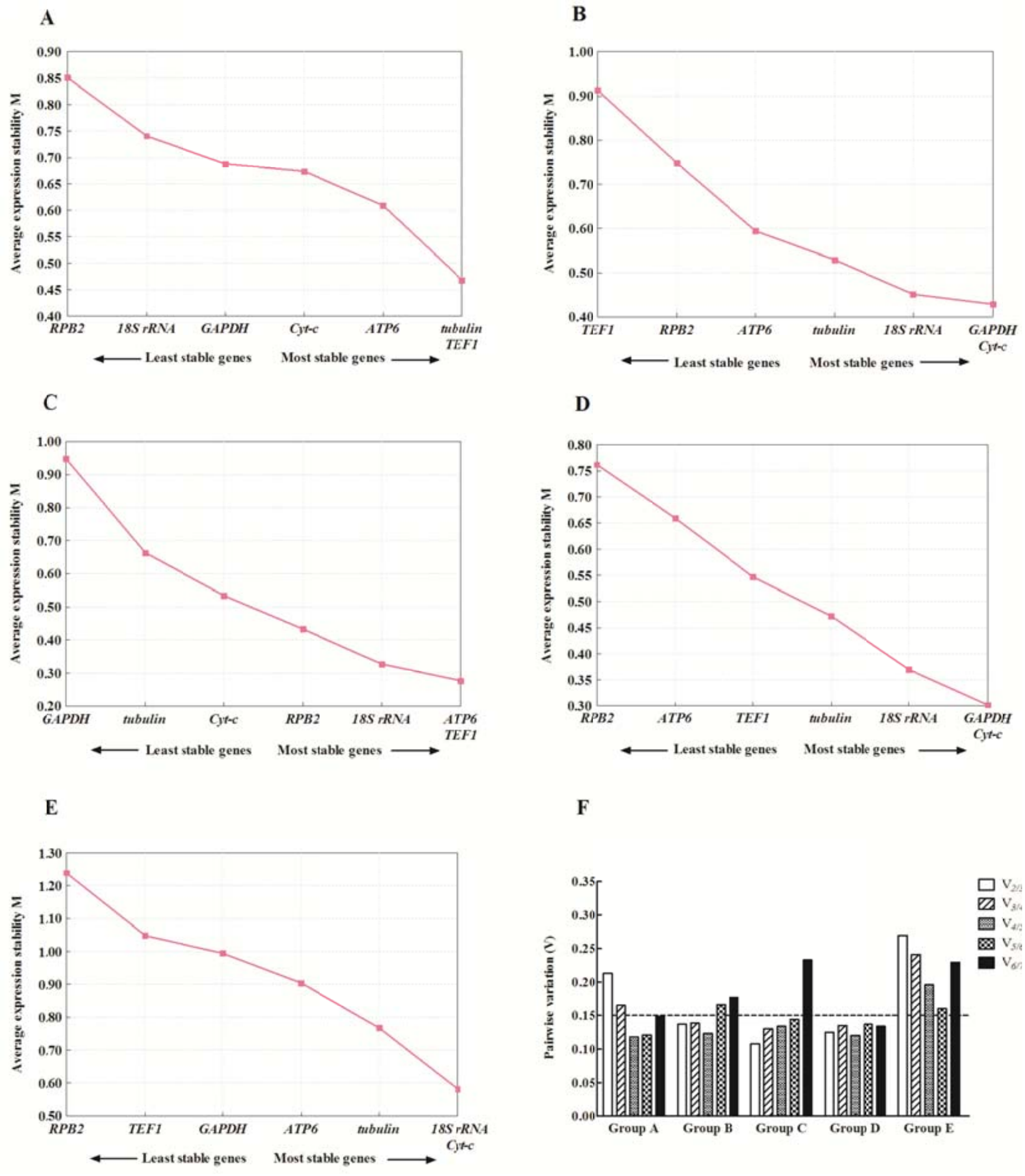

Fig. S3 Dry biomass-based laccase production of *Cerrena* sp. HYB07 in response to metal ions (A), aromatic compounds (A), carbon/nitrogen ratios (B) and nutrient types (C). Extracellular laccase activity and fungal biomass were measured on 2nd day of fermentation except for aromatic compound-treated samples. Aromatic compounds were added to the fermentation media on day 2, and measurements were performed on day 4. Samples for qPCR analysis were collected at the same time as the measurements.

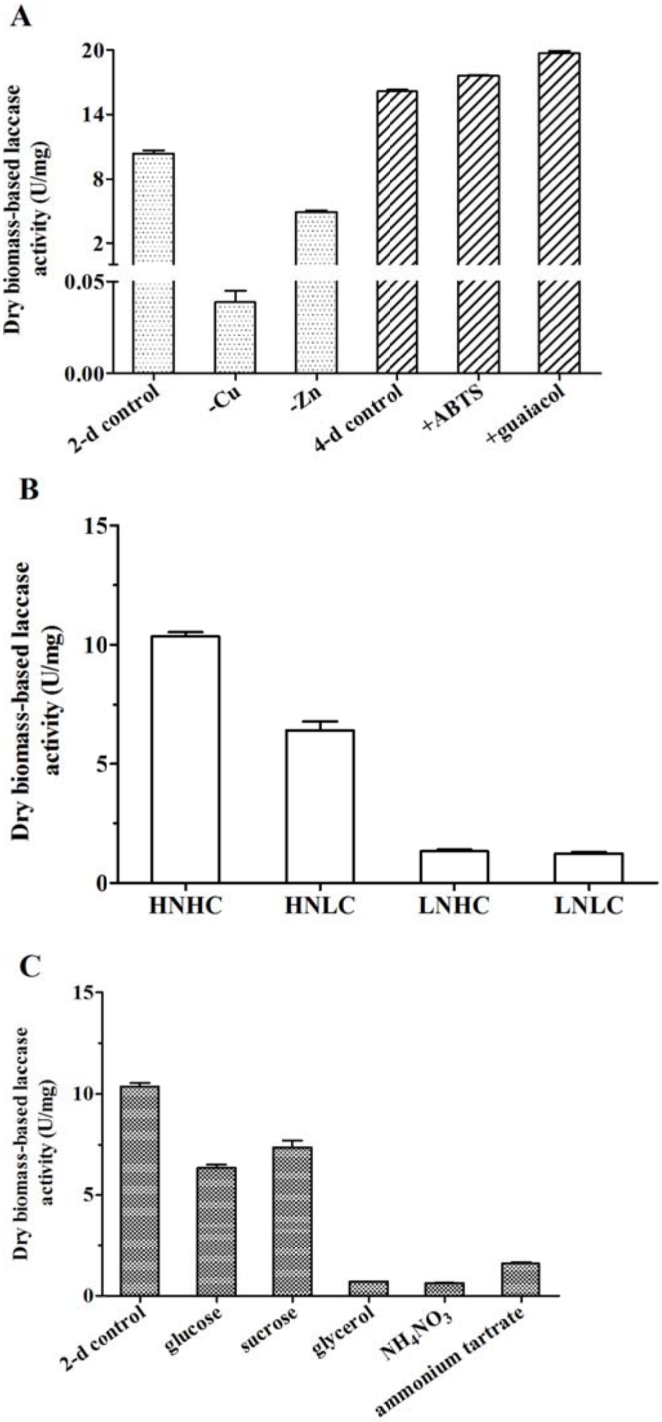

Fig. S4 Relative transcript abundance (expressed as a percentage) of the eight laccase genes under different experimental conditions.

The total transcript level of all laccase genes was taken as 100%.

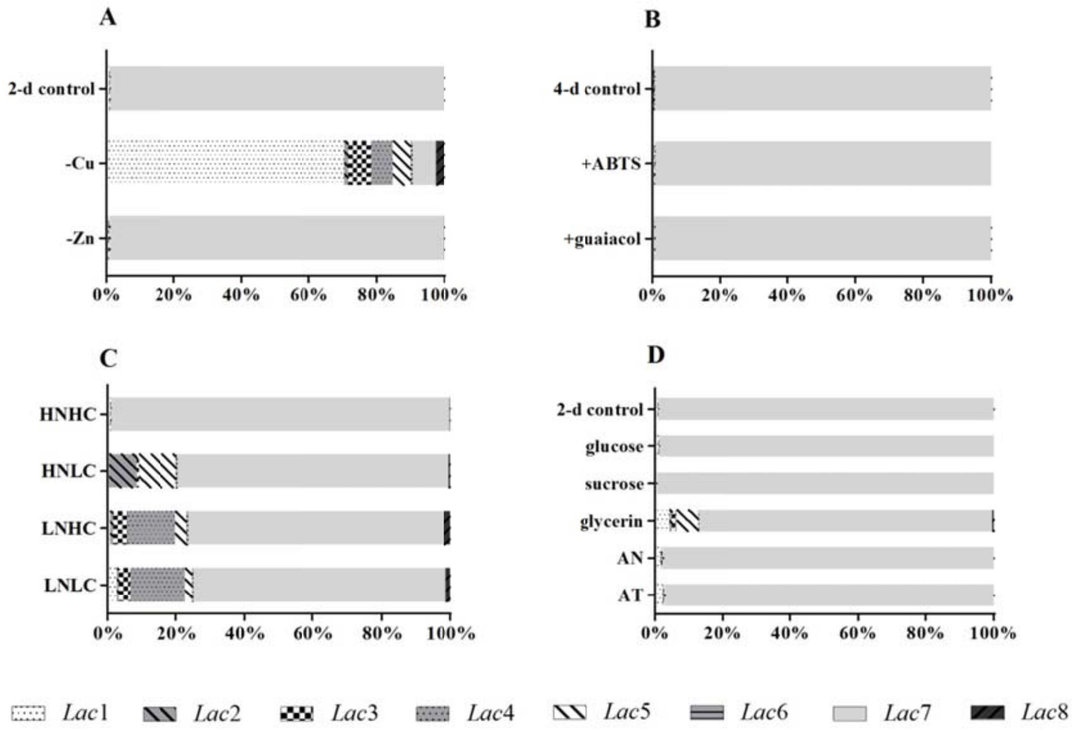

Fig. S5 Relative transcript abundance (expressed as a percentage) of the seven laccase genes after excluding the predominantly-expressed gene.

The total transcript level of the seven laccase genes was taken as 100%.

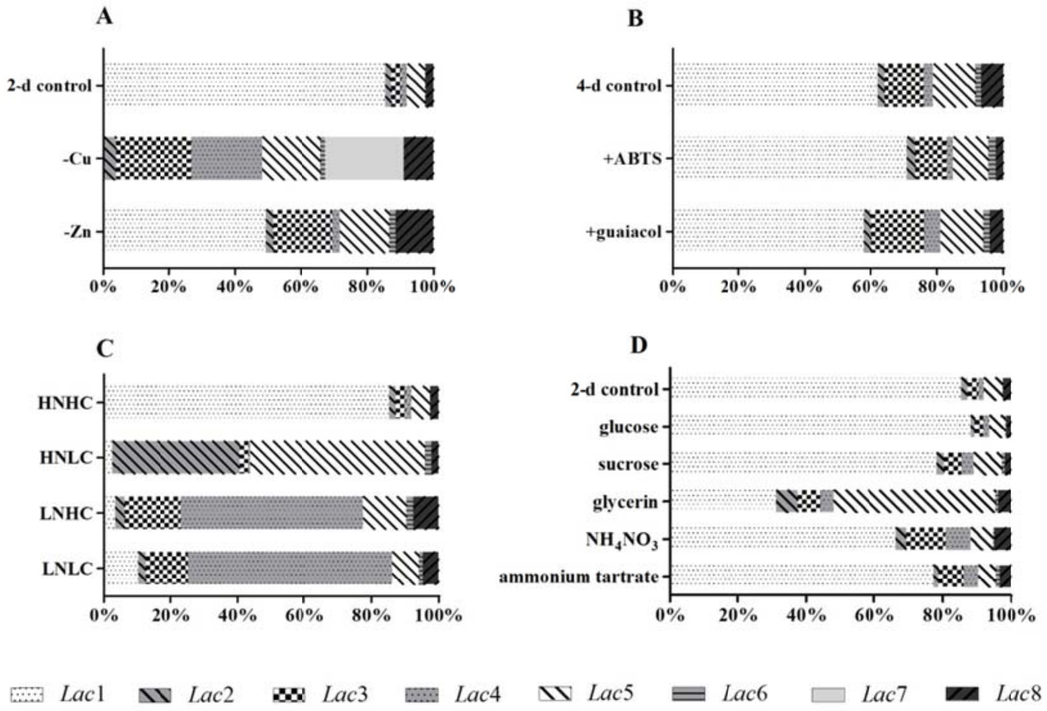

Fig. S6 *Cerrena* sp. HYB07 laccase production in the presence of aromatic compounds in PDY medium (potato dextrose broth supplemented with 0.5% yeast extract).

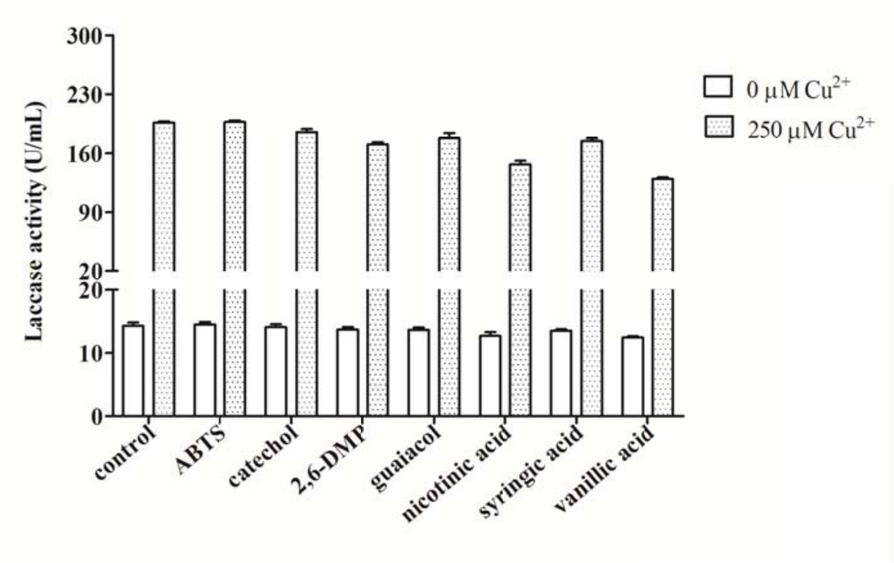

Fig. S7 Relative expression of the eight laccase genes in *Cerrena* sp. HYB07 in HNLC medium with *GAPDH* as the reference gene.

The data were also normalized with *ATP6* and *TEF1*, the validated reference genes (see Fig. 5).

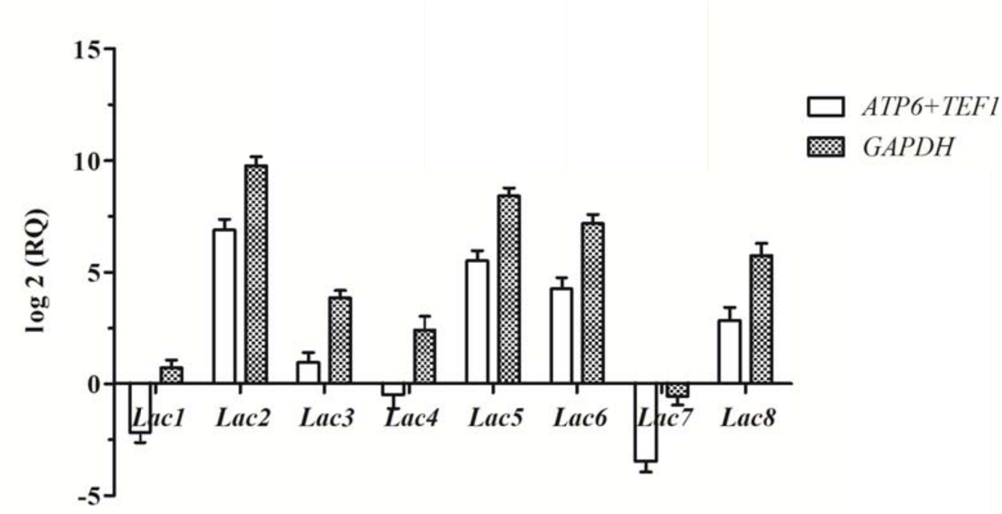

Supplement: Supplementary file 1 [file DataSheet1.pdf]
